# Socket shield technique and delayed implant placement in maxilla: a series of five case reports

**DOI:** 10.1186/s12903-022-02149-7

**Published:** 2022-04-05

**Authors:** Rola Muhammed Shadid

**Affiliations:** 1 Department of Prosthodontics, Faculty of Dentistry, Arab American University, Jenin, Palestinian Territory 240 Jenin,; 2 Prosthodontist, and Implantologist at Private Practice, Tulkarm, Palestinian Territory

**Keywords:** Socket shield, Case series, Case repot, Delayed implant placement

## Abstract

**Background:**

Tooth extraction is often followed by remodeling of hard and soft tissues, while socket shield technique has been proposed to prevent or minimize this remodeling. Socket shield accompanied with delayed implant placement is a novel technique that could be used when delayed implantation is selected; however, more scientific based evidence is required to recommend this technique as everyday clinical practice. Thus, the aim of this case series was to assess the clinical, radiographic, and esthetic outcomes of the delayed placed implants associated with previously prepared socket shield at 3–15 months follow-up after loading. The stability of the shield and the depth of soft tissue penetration palatal to the shield at reentry of 3–6 months were also assessed.

**Cases presentation:**

Five case reports of socket shield with delayed implant placement were described in the study. The facial shields were prepared and simultaneously the sockets were grafted with mineralized allograft particles, then the implants were placed 3–6 months later. Periodontal probe was used to measure the depth of soft tissue penetration palatal to the shield at reentry. Clinical indices of bleeding index, plaque index, and probing depths were recorded. Radiographic evaluation to record the amount of marginal bone loss post-loading, esthetic assessment using modified pink esthetic score, and patient assessed outcomes were also evaluated at 3–15 months follow-up after loading. At 3–6 months reentry, all shields were stable with maintenance of the facial bone and with extreme hard tissue formation in the sockets. All five implants were successful and functional without any pain or inflammation, with optimal soft tissue health and esthetics, and with minimal radiographic marginal bone loss at the last follow-up visit (3–15 months after loading).

**Conclusions:**

Within the limits of this study, socket shield technique with delayed implant placement could be a predictable minimally invasive option for cases requiring delayed implant placement; however, a long-term well-designed clinical proof is warranted.

## Background

Following tooth extraction, vertical and horizontal resorption of the alveolar ridge will usually occur mainly on the facial side in maxillary arch [1]. This is because tooth extraction is accompanied with loss of the periodontal ligament, the major source of blood supply to the facial plate [2]. In addition, the thinness of the facial bundle bone that has been demonstrated to be one millimetre or less for approximately ninety percent of patients in anterior maxilla justifies the increased susceptibility of this bone to extraction trauma and resorption [2]. This collapse of the ridge if not reversed or reduced will lead to biologic and esthetical compromise of the future restoration [3]; therefore, several suggestions and techniques have been proposed to limit the amount of this resorption. One of these techniques is the “socket shield (SS)” that was introduced in 2010 by Hurzeler and his colleagues [4]. This technique is a version of partial extraction therapy that involves extracting the whole root except the facial segment with its healthy periodontal ligament that remains attached to the facial bundle bone, and immediately the implant is placed palatal to the facial shield [4].

As a modification of Hurzeler’s socket shield, Glocker et al. [5] described a technique of socket shield but with delayed implantation. With this technique, after the socket shield is prepared, the socket is sealed with a collagen sponge to be reentered at 4–6 months later to place the implant. This approach of socket shield with delayed implantation could be used for cases where immediate implantation is not predictable or feasible due to inadequate native bone to achieve primary stability, gingival recession of the offending tooth, or due to patient’s specific factors like age, medical status, financial restraints, private or job-linked issues [6].

Although several studies were conducted on socket shield with immediate implantation and all showed promising outcomes [7–10], only one case series described the socket shield with delayed implant placement in three patients and it showed complete maintenance of the facial bundle bone at re-entry and new bone creation in the healed socket of one of the cases but without reporting on the implants follow-up [5].

Regarding the tissue pattern that develops along the palatal side of the facial shield, Pohl et al. [11] in a retrospective case series concluded that grafting the socket after shield preparation with autologous particulate dentin or tuberosity cortical bone plate prevented the soft tissue downgrowth more efficiently than PRF-grafted sockets or naturally-healing unfilled sockets.

Thus, the aim of this five-case series was to assess the clinical, radiographic, and esthetic outcomes of the delayed placed implants associated with previously prepared socket shield at 3–15 months follow-up after loading. The stability of the shield and the depth of soft tissue penetration palatal to the shield at reentry of 3–6 months were also assessed.

## Cases presentation

### Patient selection

All patients had to meet the following inclusion standards to undergo this therapy: They required at least one single implant after extraction in the maxilla from right second premolar to left second premolar with delayed implant placement protocol due to site- or patient-specific reasons like medical status, private or job-linked issues.

They had to be older than 20 years and had to be healthy without any of the following conditions: uncontrolled systemic disease like diabetes, recent chemotherapy, radiotherapy in the head or neck area, more than 10-cigarette smoking a day, intravenous bisphosphonates, pregnancy, or breastfeeding. The tooth site had to be free from any acute infection, periodontitis with > 3 mm attachment loss, ankylosis, large apical lesion that involves more than one third of root length or the lesion that will leave less than 6 mm of intact root length so the facial shield will not be stable enough to be maintained, or grade 2 or 3 mobility. All patients had to sign a written informed consent for the therapy and for publication of their cases.

Five patients of four women and a man aged 29–45 years (average, 34.6 years) presented to author’s private practice in Palestine between August 2019 and February 2021 for implant treatment. One experienced clinician (R.S) performed all the surgical and prosthetic steps.

### Treatment procedures

Standardized examination and diagnostic measures were made for all patients, consequently the treatment plan was discussed with and accepted by each patient. All patients obtained alternative treatment options with clarification of the advantages and disadvantages of each one and all of them signed a written informed consent.

All patients demonstrated very good periodontal health with low sulcus bleeding and plaque indices, and with physiologic probing depths. CBCT and Periapical radiographs were made preoperatively to evaluate the intended implant site for its suitability for socket shield technique. Scaling and oral hygiene instructions were provided to each patient two weeks prior to partial extraction, and all patients were premedicated with prophylactic 2 g amoxicillin one hour prior to surgery and 0.2% chlorhexidine mouthwash for one minute immediately pre-surgery.

After local anesthesia administration, the tooth was trimmed up to gingival margin, and all canal contents were removed by Gates Glidden drills inserted up to apical portion. The root was then sectioned mesiodistally with long shank high-speed root resection carbide bur (Komet Dental, Germany) advanced in the path created by Gates Glidden to separate the palatal segment with the apex and to maintain two third of the root length attached to the facial bundle bone. Periapical radiographs were taken meanwhile to verify the direction and extension of the bur. Once complete separation was verified, the palatal segment was removed carefully and the socket was debrided and irrigated thoroughly with sterile saline. The remaining facial shield was then trimmed to be flush with crestal bone level. Up to 1.5- to 2 mm thickness and a minimum of 6 mm length were the aimed dimensions of the facial shield. Upon completion of facial shield preparation and ensuring its stability, the socket was loosely packed with mineralized allograft particles (FDBA, Mineross, Biohorizons IPH, Birmingham, AZ, USA) and it was sealed with either a collagen sponge or platelet rich fibrin membrane (PRF) sutured to the socket margins with 5/0 polyamide nylon horizontal mattress and simple interrupted sutures (Filapeau, PETERS, France). Resin bonded acrylic bridge was used as a temporary replacement of the partially extracted tooth. Patients were instructed to continue the antibiotics (amoxicillin 500 mg) three times daily for one week, chlorhexidine gluconate 0.2% mouth wash twice daily for 2 weeks, and ibuprofen 400 mg every six hours as needed for adequate pain relief. They were recalled for evaluation post-surgery at 1, 2, 3 and 6 weeks and for suture removal at 10–14 days.

Three to six months later, a mid-crestal incision was made and a mini full thickness mucoperiosteal flap was raised facially and palatally to evaluate the bone formation in the socket and to prepare the implant site. The implant osteotomy was prepared following the manufacturer’s recommendations to place the implant in the correct 3D position palatal to facial shield. The implants (AnyRidge MegaGen, MegaGen Implant Co., Ltd., South Korea, 5° Morse Taper connection) were then inserted and insertion torque (IT), implant stability quotient (ISQ) values (MegaISQ; MegaGen Implant Co., Ltd), and periapical radiographs were obtained for each implant. All implants had a minimum of 25 N/cm IT and a minimum of 65 ISQ, so a customized healing abutment was attached to each one. A single dose of 2gm amoxicillin was taken preoperatively, and chlorhexidine mouthwash and nonsteroidal anti-inflammatory drug were prescribed as previously mentioned in the first surgery. After approximately 3 months, the customized abutment was removed and the ISQ was recorded to start with the definitive prosthetic phase. All implants had ≥ 70 ISQ, so a customized pick-up impression coping was attached to the implant and the impression was made with putty soft/light body addition silicone material (Elite HD+, Zhermack SpA, Italy). Screw-retained zirconia crowns were delivered to all patients with the screw torqued to 35 N/Cm using a calibrated torque wrench (MegaGen Implant Co., Ltd., South Korea). Teflon tape and bulk fill composite material (Palfique Bulk Flow, Tokuyama Dental Corporation, Japan) sealed the screw access holes and a periapical radiograph was made as a baseline. The characteristics and details of the five cases are presented in Table 1.

**Table 1:** Cases description, implant dimensions, and follow-up time

Patient no	Age (years)	Sex	Tooth type	Extraction cause	Facial plate thickness (mm)	Gingival biotype	Smile line	Socket reentry time (months)	Inserted implant (AnyRidge)	Loading time (months)	Overall follow-up (months)
1	31	M	2^nd^ premolar	Deep subgingival caries	1	Thick	Low	5	3.5 × 15	15	19
2	29	F	2^nd^ premolar	Deep subgingival caries	0.9	Thick	Low	6	3.5 × 11.5	13	17
3	45	F	Canine	Horizontal fracture	1	Thick	High	3	3.5 × 15	3	5
4	30	F	Lateral incisor	Horizontal fracture	1	Thick	Average	4	3.5 × 15	5	8
5	38	F	Canine	Horizontal fracture	1.1	Thick	Low	4	3.5 × 13	9	12

### Patient 1

**Fig. 1:**
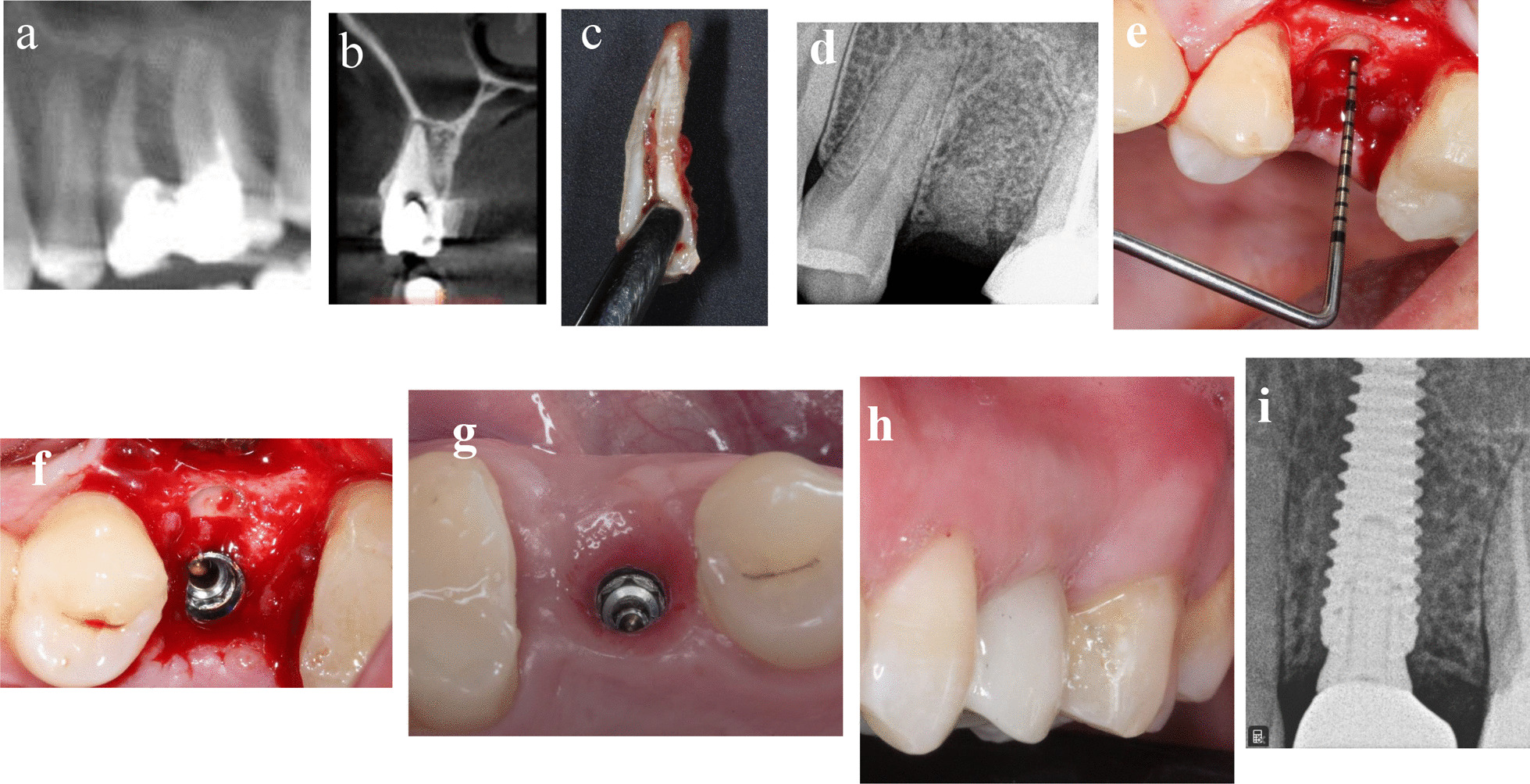
**a**–**i** Photos and radiographs of case #1 replacing maxillary left second premolar. **a** Panoramic view of preoperative CBCT; **b** cross-sectional view of preoperative CBCT; **c** the extracted palatal portion with the apex; **d** periapical radiograph immediately after shield preparation and bone grafting; **e** the depth of soft tissue penetration measured with periodontal probe; **f** implant placed palatal to the shield in healed socket; **g** occlusal view of the healed peri-implant soft tissue; **h** frontal view of the final implant restoration at 15 months post-loading; **i** periapical radiograph at 15 months post-loading

A 31-year-old-male medically fit non-smoker patient presented in September 2019 with a deep subgingival caries and failed endodontic treatment of maxillary left second premolar (Fig. 1a–i). The patient requested a definitive restoration of this tooth. After discussing with the patient different treatment options like endodontic retreatment and orthodontic forced eruption or crown lengthening to allow the tooth to be crowned with sufficient ferrule, or single tooth implant crown, the patient selected the latter option and consented the socket shield technique with delayed implant placement since the cross sectional CBCT showed insufficient native bone to achieve good primary stability for immediate implant placement. After 5 months of shield preparation and socket grafting as previously discussed in the “treatment procedures” section, osteotomy was prepared to receive 3.5 × 15 mm implant (AnyRidge, Megagen) with ISQ 65/67. Customized healing abutment was placed and 3 months later, ISQ raised to ≥ 70 and screw-retained zirconia crown was delivered. The patient was followed up to 15 months after loading.

### Patient 2

**Fig. 2:**
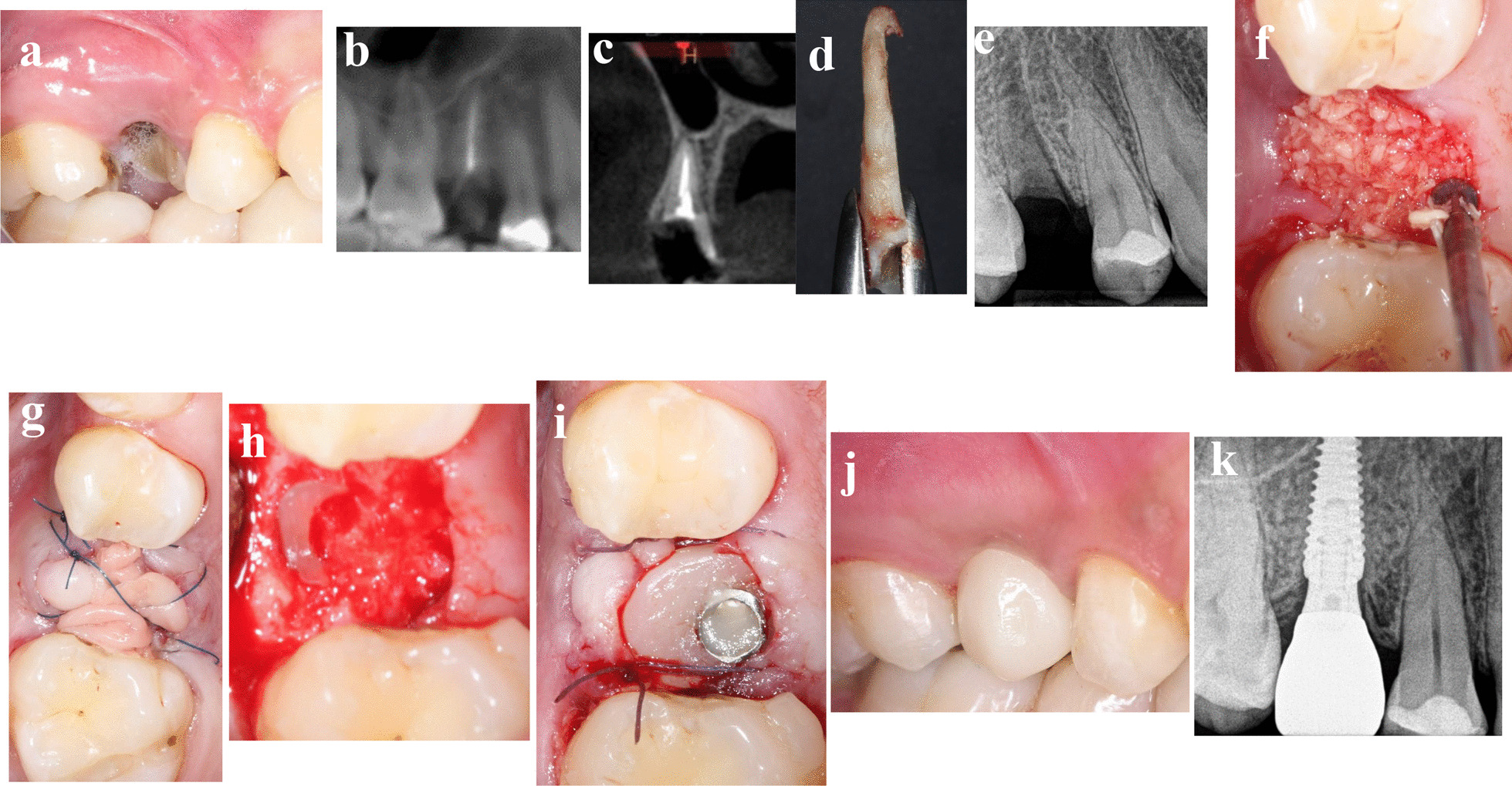
**a**–**k** Photos and radiographs of case #2 replacing maxillary right second premolar. **a** Preoperative buccal view; **b** panoramic view of preoperative CBCT; **c** cross-sectional view of preoperative CBCT; **d** the extracted palatal portion with the apex; **e** periapical radiograph after shield preparation; **f** FDBA particles filled the socket; **g** PRF membrane sealed the socket; **h** the healed socket at reentry of 6 months; **i** customized healing abutment fitted immediately after implant placement; **j** buccal view of the final implant restoration at 13 months post-loading; **k** periapical radiograph at 13 months post-loading

A 29-year-old-female medically fit non-smoker patient presented in August 2019 with non-restorable maxillary right second premolar and asked for implant therapy to replace this tooth (Fig. 2a–k). Since the cross sectional CBCT showed insufficient native bone to achieve good primary stability for immediate implant placement, two implant therapy options were presented to the patient, either conventional delayed implant placement (3–4 months postextraction) or socket shield technique followed by delayed implantation. The patient accepted the socket shield delayed implantation. Six months after shield preparation, 3.5 × 11.5 mm implant (AnyRidge, Megagen) with ISQ 75/70 was placed, and all surgical and prosthetic procedures were performed as previously discussed in the “treatment procedures” section. The patient was followed up to 13 months after loading.

### Patient 3

**Fig. 3:**
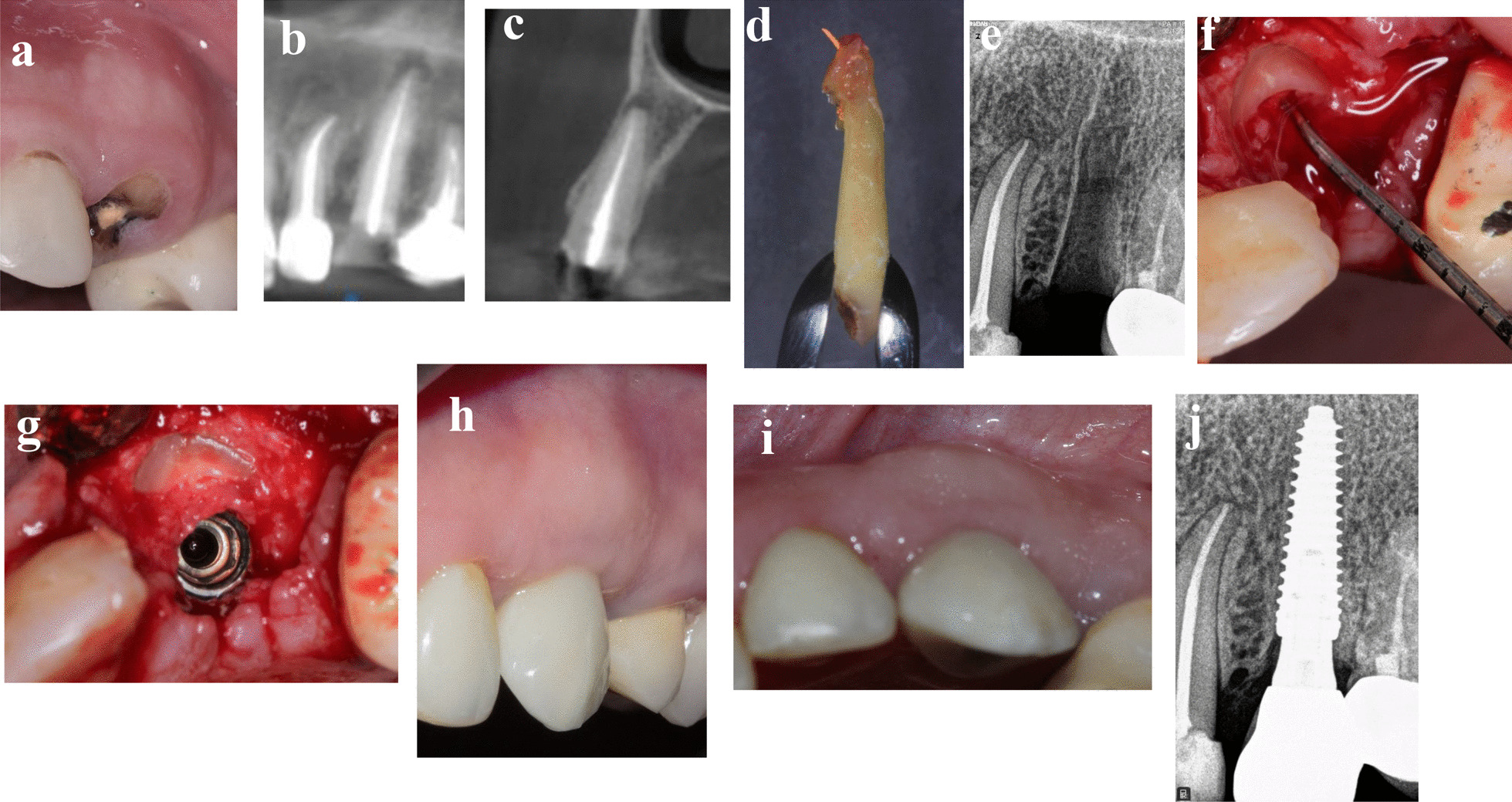
**a**–**j** Photos and radiographs of case #3 replacing maxillary left canine. **a** Preoperative frontal view; **b** panoramic view of preoperative CBCT; **c** cross-sectional view of preoperative CBCT; **d** the extracted palatal portion with the apex; **e** periapical radiograph after shield preparation; **f** the depth of soft tissue penetration measured with periodontal probe; **g** implant placed palatal to the shield in healed socket; **h** frontal view of the of the final implant restoration at 3 months post-loading; **i** incisal view of the of the final implant restoration at 3 months post-loading; **j** periapical radiograph at 3 months post-loading

A 45-year-old-female medically fit non-smoker patient presented in February 2021with fractured maxillary left canine at gingival level with deep subgingival caries (Fig. 3a–j). The patient requested a replacement of this fractured tooth. After thorough clinical and radiographic examination, different treatment options were offered to the patient including orthodontic forced eruption, conventional immediate implant, socket shied with immediate implant, and socket shield followed by delayed implant placement. Socket shield with delayed implantation was selected. Three months after shield preparation, 3.5 × 15 mm implant (AnyRidge, Megagen) with ISQ 65/67 was placed, and all surgical and prosthetic procedures were performed as previously discussed in the “treatment procedures” section. The patient was followed up to 3 months after loading.

### Patient 4

**Fig. 4:**
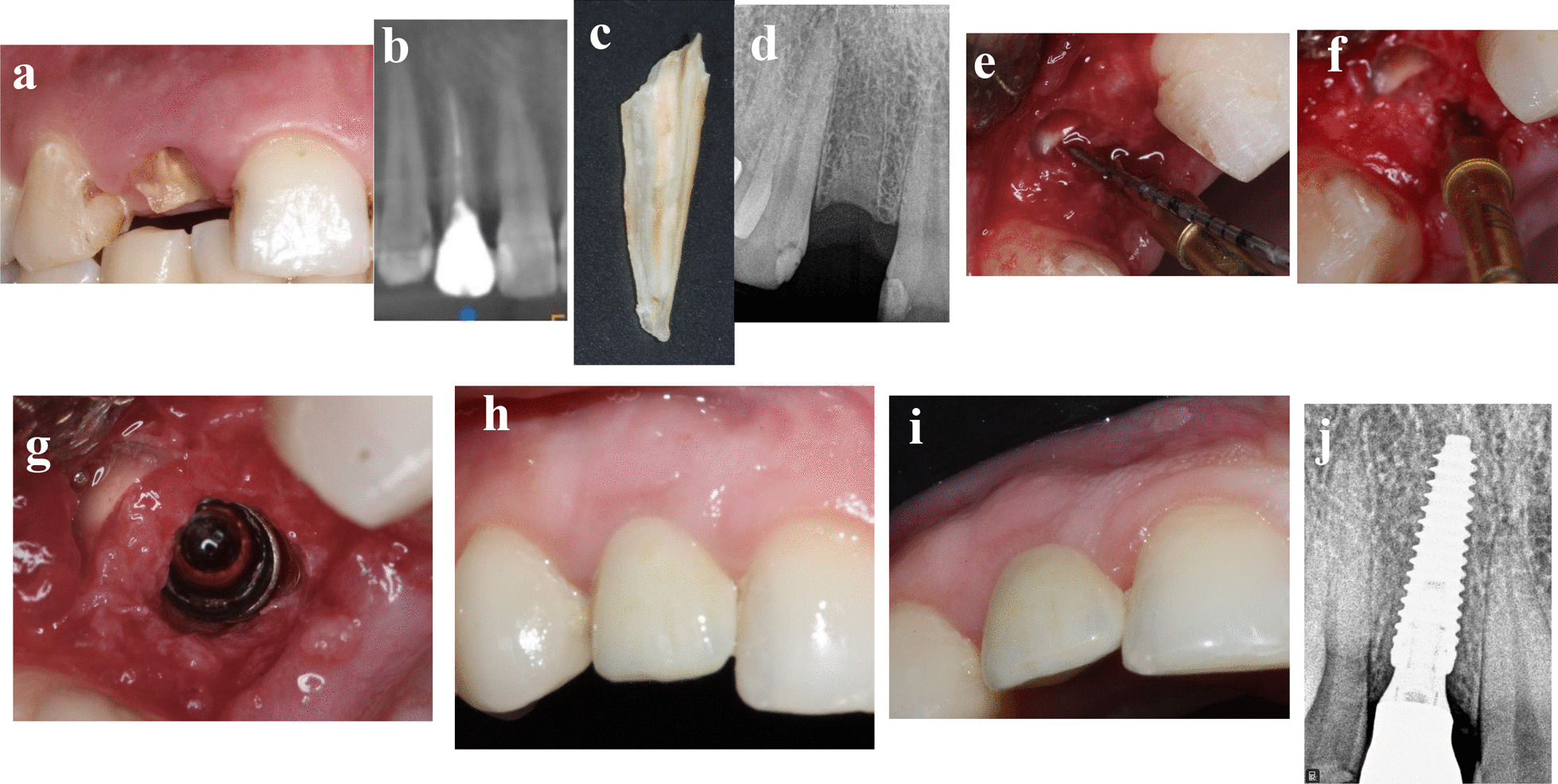
**a**–**j** Photos and radiographs of case #4 replacing maxillary right lateral incisor. **a** Preoperative frontal view; **b** panoramic view of preoperative CBCT; **c** the extracted palatal portion; **d** periapical radiograph after shield preparation; **e** the depth of soft tissue penetration measured with periodontal probe; **f** osteotomy preparation in healed socket palatal to the shield; **g** implant placed palatal to the shield; **h** frontal view of the of the final implant restoration at 5 months post-loading; **i** incisal view of the of the final implant restoration at 5 months post-loading; **j** periapical radiograph at 5 months post-loading

A 30-year-old-female medically fit non-smoker patient was seeking implant therapy to substitute her fractured maxillary right lateral incisor in October 2020 (Fig. 4a–j). The cervical fracture of the tooth with lack of ferrule made it unrestorable unless orthodontic forced eruption would be made. The patient refused orthodontic extrusion of the tooth and asked for implant therapy. Conventional immediate implant, socket shield immediate implant, and socket shield in conjunction with delayed implant placement were the presented treatment options to replace this tooth, while the patient consented for socket shield with delayed implant placement. Four months after shield preparation, 3.5 × 15 mm implant (AnyRidge, Megagen) with ISQ 69/68 was placed, and all surgical and prosthetic procedures were performed as previously discussed in the “treatment procedures” section. The patient was followed up to 5 months after loading.

### Patient 5

**Fig. 5:**
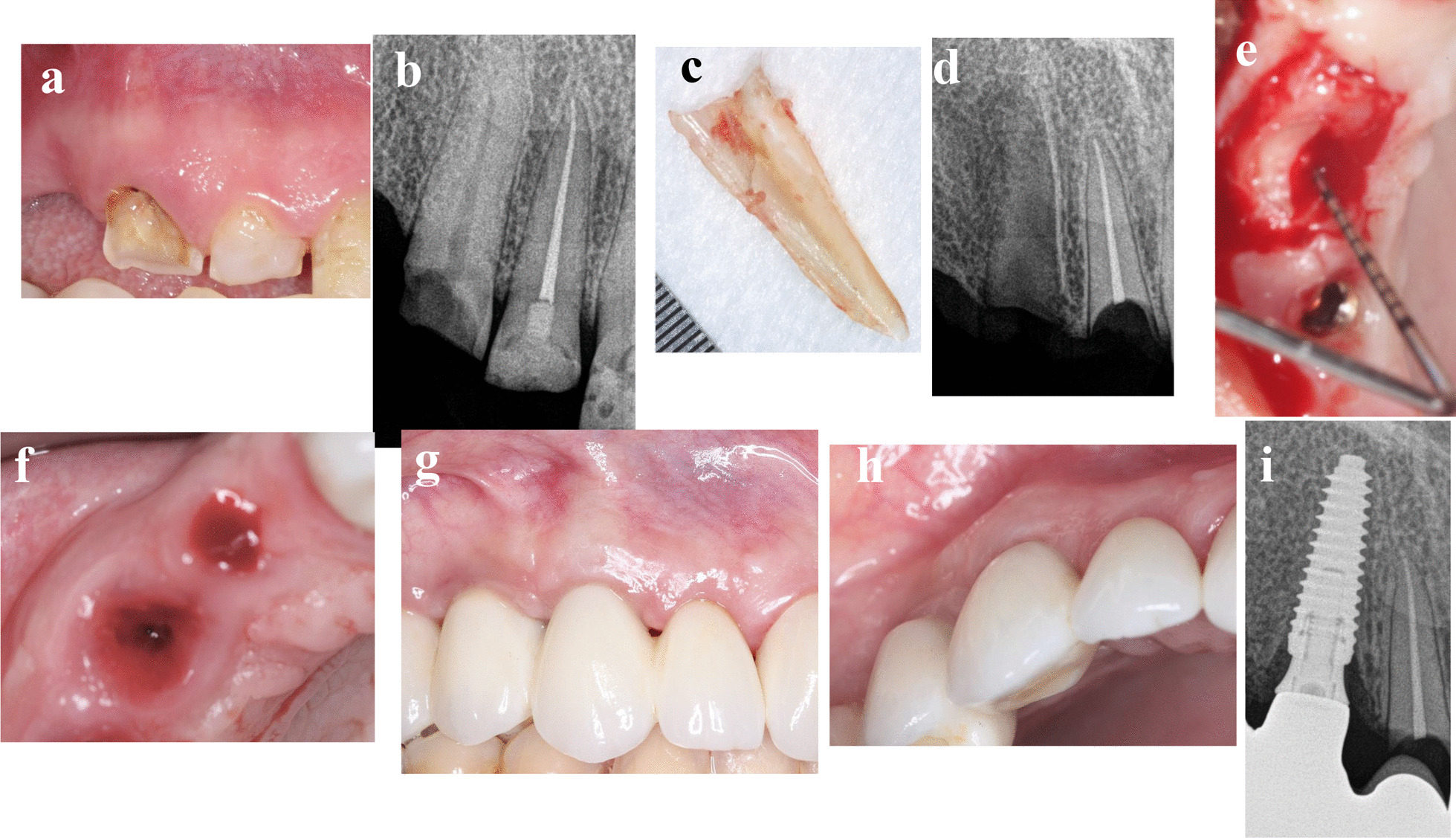
**a**–**i** Photos and radiographs of case #5 replacing maxillary right canine. **a** Preoperative frontal view; **b** preoperative periapical radiograph; **c** the extracted palatal portion; **d** periapical radiograph after shield preparation and submergence of lateral incisor root; **e** the depth of soft tissue penetration measured with periodontal probe; **f** incisal view of peri-implant soft tissue at 4 months post-implantation; **g** frontal view of the final implant restoration at 9 months post-loading; **h** incisal view of the of the final implant restoration at 9 months post-loading; **i** periapical radiograph at 9 months post-loading

A 38-year-old-female medically fit non-smoker patient was referred to author’s private practice from a colleague in February 2020 to replace her fractured maxillary right canine and the adjacent lateral incisor with an implant-supported restoration (Fig. 5a–i). After thorough examination and discussion with the referring dentist and with the patient, option of socket shield with delayed implant placement in the canine area and root submergence of lateral incisor was accepted by both the dentist and the patient, while the dentist would continue the treatment of the maxillary arch according to a comprehensive treatment plan for full maxillary arch rehabilitation. According to the same protocol followed in the previously mentioned cases, implant of 3.5 × 13 mm (AnyRidge, Megagen) with ISQ of 75/70 was placed 4 months after shield preparation, and all surgical and prosthetic procedures were performed as previously discussed in the “treatment procedures” section. A definitive 5-unit screw-retained zirconia bridge supported by right canine, right first premolar, and right first molar implants with cantilevered lateral incisor pontic was delivered. The patient was followed up to 9 months after loading.

### Evaluation of the socket shield at reentry (3–6 months)

To measure the depth of soft tissue penetration at the palatal side of the facial shield at reentry (Table 1), a 15-mm manual periodontal probe (Hu-Friedy) was firmly advanced in the junction between the palatal side of the facial shield and the surrounding area. The depth of soft tissue penetration was recorded to the nearest millimeter from the most coronal portion of the shield up to the first apical hard tissue touched. A clinical photograph documenting this penetration was captured [11].

### Clinical and radiographic evaluation of inserted implants

Sulus bleeding index (SBI) [12], modified plaque index (PI) [13], and probing depth (PD) were evaluated at mesial, facial, distal, and palatal sites of the implant restoration at delivery and at the last follow-up visit (3–15 months after loading). Light probing load of nearly 25 g was used to evaluate the probing depth to the nearest millimeter using 15-mm manual periodontal probe (Hu-Friedy). For each index, one value was recorded based on the average of the four attained values [14]. The width of facial keratinized mucosa was also recorded for all cases.

To analyze the mesial and distal marginal bone level changes from restoration delivery until the last follow-up appointment 3–15 months post-loading (Table 1), standardized digital periapical radiographs (Carestream, USA) using the parallax technique with Kerr sensor holders (Kerr Dental, USA) were made at implant insertion, at crown delivery, and at last follow-up visit. The radiographic distance from the implant shoulder to the first bone-implant contact mesially and distally was measured digitally with a software (CS Imaging Software -7.0.3, USA). The marginal bone coronal to implant shoulder was considered as 0.0 to simplify the process. For calibration, the distance between the tips of the fixture threads of 0.8 mm was used as reference.

### Esthetic evaluation

Modified pink esthetic score (mPES) was used to assess the peri-implant soft tissue esthetics evaluating five items: mesial papilla, distal papilla, level of the facial peri-implant soft tissue, curvature of the facial peri-implant soft tissue, and root convexity/soft tissue color and texture at the facial aspect of the implant site [15]. These parameters were evaluated using digital photographs taken at the last follow-up visit (3–15 months after loading) with DSLR camera (Canon EOS 60 D, Tokyo, Japan, 100-mm Canon macro lens with ring flash). A record of 0,1, or 2 was allocated to all five PES items with a score of 10 being the highest esthetic score.

### Patient-assessed outcomes

At one month after delivery of the final restoration, the patients were invited to answer the following questions (Qs) focusing on patient’s satisfaction with the final restoration, the peri-implant soft tissue, and the overall treatment [16].

- Q1) From 0 to 10, how would you value your satisfaction regarding the final implant restoration?
- Q2) From 0 to 10, how would you value your satisfaction regarding the peri-implant soft tissue?
- Q3) From 0 to 10, how would you value your satisfaction regarding the overall treatment?

### Implant success assessment

Implant success was assessed according to the Smith and Zarb success criteria [17]. The implant was regarded as a failure if it showed notable mobility, peri-implant radiolucency, significant vertical bone loss more than 1.5 mm in the first year of loading and more than 0.2 mm yearly after the first year of loading, persistent pain, infection, or unacceptable prosthetic position.

### Findings

Concerning the stability of the facial shield at reentry 3–6 months post-preparation, all five cases demonstrated stable shields with preservation of the facial bone, while the depth of soft tissue penetration palatal to shield was about 1 mm in four cases and 3 mm in one case (Table 2).

**Table 2:** Results of depth of soft tissue penetration, clinical indices, and peri-implant marginal bone loss

Pt. no	DSTP (mm)	Clinical indices			MBL mesial (mm)	MBL distal (mm)
		SBI	PI	PD		
1	1	0.5	0.5	1.25	0.0	0.0
2	1	0.25	0	1.5	0.0	0.0
3	1	0.0	0.25	1.25	0.0	0.0
4	1	0.5	0.5	2.0	0.0	0.0
5	3	0.25	0.0	2.0	0.32	0.56

Pt: patient; DSTP: depth of soft tissue penetration; SBI: sulcus bleeding index; PI: modified plaque index;

PD: pocket depth; MBL: marginal bone loss

At the last follow-up visit (3–15 months after loading), the soft tissue looked healthy without any sign of peri-implant mucositis. All cases exhibited low sulcus bleeding and plaque indices, and physiologic probing depths (Table 1). Three-to-five-millimeter width of facial keratinized mucosa was recorded for all cases.

Radiographic evaluation of the five implants did not show any sign of continuous radiolucency throughout the follow-up period. Four implants exhibited no marginal bone loss from restoration delivery until the last follow-up visit (3–15 months after loading), provided that these implants had marginal bone level coronal to the implant platform and it remained the same till the last recall. However, one implant showed an average marginal bone loss of 0.44 mm in the first year of loading (Table 2).

Regarding the esthetic outcomes, all cases achieved modified pink esthetic scores of 8 or more, representing optimal soft tissue esthetic results (Table 3). Further, all patients expressed excellent satisfaction with the esthetic outcomes of the final restoration and the peri-implant mucosa, and with the overall treatment (Table 3).

**Table 3:** Results of modified pink esthetic scores, patient-assessed outcomes, and complications

Pt. no	mPES	Patient-assessed outcomes			Complications
		Q1	Q2	Q3	
1	9	10	10	9	Nil
2	10	10	10	10	Nil
3	8	9	10	10	Nil
4	9	10	10	10	Nil
5	9	10	10	10	Nil

Pt: patient; mPES: modified pink esthetic score; Q1: patient appreciation of implant restoration; Q2: patient appreciation of peri-implant soft tissue; Q3: patient appreciation of overall treatment

With regard to the implant success assessment, all implants healed uneventfully without any complications and with ISQ elevated up to ≥ 70 in the three months healing period. Until the last follow-up appointment, all of them were stable and functional without any pain or inflammation. Radiographically, there was no any peri-implant radiolucency or peri-implant marginal bone loss greater than 1.5 mm in the first year of loading as presented in Table 2.

## Discussion and conclusion

The present study described a series of five cases in which the socket shield was prepared according to the most recently proposed guidelines [18]. Mineralized allograft particles were used to graft the socket to prevent soft tissue ingrowth by its hard structures and at the same time to resorb at a reasonable controlled rate compatible with the intended timing of implant placement.

To the best of the author’s knowledge, this study is the first case series to report on the clinical, radiographic, esthetic, and patient-assessed outcomes of delayed placed implants accompanied with socket shield at 3–15 months follow-up after loading. The depth of soft tissue penetration palatal to the shield at 3–6 months post-preparation was also assessed. The reported cases showed that the facial shields were stable at reentry of 3–6 months post-preparation with preservation of the facial bone. This is in agreement with a previously published case series study [5]. Concerning the depth of soft tissue penetration palatal to the shield at reentry, it was about 1 mm in four cases and 3 mm in another case. This is supported by the results of a recent retrospective case series [11] that showed that the sockets with prepared facial shields healed with substantial soft tissue ingrowth when allowed to heal unassisted, while similar sockets when filled with autologous particulate dentin or cortical tuberosity bone plate healed with only 1 mm soft tissue ingrowth depth and with extreme hard tissue formation. The reduced bone formation in the unfilled sockets could be attributed to the presence of a root shield that impedes the migration of the osteoprogenitor cells into the socket to form new bone [19]. Regarding the available human histologic evidence on the nature of the intervening tissue between the shield and the implant, only two case reports were published [20, 21]. A human histologic analysis of a retrieved implant five years after immediate placement with socket shield showed mature bone interface between the shield and the implant in the apical and middle thirds while soft tissue colonized in the coronal third [20]. Another human histologic investigation revealed that mature bone occupied the space between the root dentin and the implant threads after two years of implant placement with unplanned socket shield [21].

This study showed that all implants were successful and functional without any pain or inflammation and with optimal soft tissue health and esthetics at 3–15 months follow-up after loading. Radiographically, there was no any peri-implant radiolucency or more than normal bone remodeling till the last recall time. In the literature, only one case series of three patients evaluated socket shield with delayed implant placement. It showed complete maintenance of the facial bundle bone at reentry and new bone creation in the healed socket of one of the cases that was re-entered at six months for implantation [5]; however, it did not report on any follow-up of implant survival or complications. On the other hand, several studies were conducted on socket shield immediate implant placement and all showed promising results [7–10]. A retrospective case series study [22] that evaluated the clinical, radiographic, and volumetric changes that occurred after 5 years of ten socket shield immediate implant placement in the maxilla showed that all implants survived with minimal bone loss, optimal esthetic outcomes, and with effective preservation of peri-implant facial contours.

Although this case series demonstrated that all implants were successful without any complication and with optimum esthetic results, it presents with some limitations including small sample size with limited variables and with relatively short follow-up. Therefore, to recommend this technique as routine treatment, well-designed randomized and prospective clinical studies with long observation periods have to be conducted. In addition, to know more about the intervening tissues between the shield and the implant, histologic investigations are recommended.

Within the limitation of analysis of a five-case series, socket shield technique with delayed implant placement could be a predictable minimally invasive option for cases demanding delayed implant placement. At 3–6 months reentry, all shields were stable with maintenance of the facial bone and with extreme hard tissue formation in the sockets allowing to place the implants in the correct 3D position with high primary stability. All implants were successful and functional without any pain or inflammation and with optimal soft tissue health and esthetics at the last follow-up visit (3–15 months after loading). Radiographically, there was no any peri-implant radiolucency or more than normal peri-implant marginal bone remodeling in the first year of loading.

## Data Availability

Data and materials can be obtained upon request by sending an email to rola.shadid@aaup.edu.

## Abbreviations

DSLR: Digital single-lens reflex camera
FDBA: Freeze-dried bone allograft
ISQ: Implant stability quotient
CBCT: Cone-beam computed tomography
PRF: Platelet rich fibrin membrane
